# Atomic Weights

**Published:** 1891-04

**Authors:** W. H. Whitslar


### Atomic Weights.

W. H. WHITSLAR, D.D.S.

Chemistry may now be said to be an exact science, because of the fixed values of chemical bodies and the many uncontrovertable facts. In the matter of valuation, however, the Committee of Revision and Publication of the Pharmacopeoia of the United States have deemed a modification necessary, and accordingly, Professor F. W. Clark, Chief Chemist of the U. S. Geological Survey, has furnished a table of atomic weights revised upon the basis of the most recent data and his latest computations. All calculations and analytical data which are to be given in reports or contributions intended for use by the committee, must be based upon these values. In speaking of the matter, the *Medical Record* recommend the adoption in general by chemists of the revised table. Not that a difference of a fraction in atomic weight of an element would materially affect the composition of a medicinal compound, but it is for the sake of unity that the table should be generally adopted. Professor Clark has at his command every requisite and convenience for his work, and his authority is unquestioned.

The following table is a part of the table of weights as authorized, and will be found of interest to the dentist as well as the chemist. This table includes the official elements.

| NAME. | Symb'l | Atomic Weight. | NAME. | Symb'l | Atomic Weight. |
|---|---|---|---|---|---|
| Aluminium | Al. | 27. | Iron | FE. | 56. |
| Antimony | Sb. | 120. | Lead | Pb. | 206.95 |
| Arsenic | As. | 75. | Lithium | Li. | 7.02 |
| Barium | Ba. | 137. | Magnesium | Mg. | 24.3 |
| Bismuth | Bi. | 208. | Manganese | Mn. | 55. |
| Boron | B. | 11. | Mercury | Hg. | 200. |
| Bromine | Br. | 79.95 | Nitrogen | N. | 14.03 |
| Calcium | Ca. | 40. | Oxygen | O. | 16. |
| Carbon | C. | 12. | Phosphorus | P. | 31. |
| Cerium | Ce. | 140.2 | Potassium | K. | 39.11 |
| Chlorine | Cl. | 35.45 | Silicon | Si. | 28.4 |
| Chromium | Cr. | 52.1 | Silver | Ag. | 107.92 |
| Copper | Cu. | 63.4 | Sodium | Na. | 23.05 |
| Gold | Au. | 197.3 | Sulphur | S. | 32.06 |
| Hydrogen | H. | 1.007 | Zinc | Zn. | 65.3 |
| Iodine | I. | 126.85 | | | |

From the above it will be seen by comparison with the table of atomic weights to be found in the last ( 15th ) edition of the United States Dispensatory, that no less then twenty-two of the above elements have been changed in their molecular or atomic weight. Notably are the following: Chlorine, 35.4, now 35.45; Gold, 196.2, now 197.3; Hydrogen 1, now 1.007; Iron 55.9, now 56; Lead 206.5, now 206.95; Mercury 199.7, now 200; Potassium 39, now 39.11; Silver 107.7, now 107.92; Zinc 64.9, now 65.3, etc.

Now this difference would not practically make any difference with a medical compound, but it is for the sake of uniformity that all are requested to adopt the revision, thus saving much labor in problems of chemical equivalence.

